# Genetics, Structure, and Function of Group A Streptococcal Pili

**DOI:** 10.3389/fmicb.2021.616508

**Published:** 2021-02-09

**Authors:** Masanobu Nakata, Bernd Kreikemeyer

**Affiliations:** ^1^Department of Oral Microbiology, Graduate School of Medical and Dental Sciences, Kagoshima University, Kagoshima, Japan; ^2^Institute of Medical Microbiology, Virology and Hygiene, University of Rostock, Rostock, Germany

**Keywords:** *Streptococcus pyogenes*, pili, thermoregulation, T serotyping, FCT region

## Abstract

*Streptococcus pyogenes* (Group A *Streptococcus*; GAS) is an exclusively human pathogen. This bacterial species is responsible for a large variety of infections, ranging from purulent but mostly self-limiting oropharynx/skin diseases to streptococcal sequelae, including glomerulonephritis and rheumatic fever, as well as life-threatening streptococcal toxic-shock syndrome. GAS displays a wide array of surface proteins, with antigenicity of the M protein and pili utilized for M- and T-serotyping, respectively. Since the discovery of GAS pili in 2005, their genetic features, including regulation of expression, and structural features, including assembly mechanisms and protein conformation, as well as their functional role in GAS pathogenesis have been intensively examined. Moreover, their potential as vaccine antigens has been studied in detail. Pilus biogenesis-related genes are located in a discrete section of the GAS genome encoding fibronectin and collagen binding proteins and trypsin-resistant antigens (FCT region). Based on the heterogeneity of genetic composition and DNA sequences, this region is currently classified into nine distinguishable forms. Pili and fibronectin-binding proteins encoded in the FCT region are known to be correlated with infection sites, such as the skin and throat, possibly contributing to tissue tropism. As also found for pili of other Gram-positive bacterial pathogens, GAS pilin proteins polymerize via isopeptide bonds, while intramolecular isopeptide bonds present in the pilin provide increased resistance to degradation by proteases. As supported by findings showing that the main subunit is primarily responsible for T-serotyping antigenicity, pilus functions and gene expression modes are divergent. GAS pili serve as adhesins for tonsillar tissues and keratinocyte cell lines. Of note, a minor subunit is considered to have a harpoon function by which covalent thioester bonds with host ligands are formed. Additionally, GAS pili participate in biofilm formation and evasion of the immune system in a serotype/strain-specific manner. These multiple functions highlight crucial roles of pili during the onset of GAS infection. This review summarizes the current state of the art regarding GAS pili, including a new mode of host-GAS interaction mediated by pili, along with insights into pilus expression in terms of tissue tropism.

## Introduction

Several different types of pathogenic bacteria colonize distinct niches by adhering to host tissues via long filamentous appendages termed pili or fimbriae, which project from the cell surface. Pili are also involved in conjugation, twitching motility, and virulence. Gram-positive bacterial pili were undetected until recently because of their thin structure. Following their discovery in bacteria belonging to the *Actinomyces* and *Corynebacterium* genera (Yanagawa and Honda, 1976; Cisar and Vatter, 1979), and the unraveling of their assembly mechanisms (Ton-That and Schneewind, 2003), pili of pathogenic streptococci, including *Streptococcus agalactiae*, *Streptococcus pyogenes*, and *Streptococcus pneumoniae* have been reported since 2005 (Lauer et al., 2005; Mora et al., 2005; Barocchi et al., 2006). Commensal oral streptococci, such as *Streptococcus sanguinis* and *Streptococcus oralis*, were also shown to produce pili (Okahashi et al., 2010; Zähner et al., 2011). Pili are considered to be physiologically distinctive to typical cell wall-anchored surface proteins regarding biological functions during the course of infection, since covalent linkage of subunits allows pilus proteins to locate not only on the bacterial cell surface but also >1 μm away from the surface, thus providing first contact with host molecules.

*Streptococcus pyogenes* (Group A *Streptococcus*; GAS) is a human pathogen responsible for a wide variety of human diseases (Walker et al., 2014). The major manifestations of GAS infections are local suppurative inflammation in the upper respiratory tract and skin, i.e., pharyngitis and impetigo. The annual number of pharyngitis cases worldwide has been estimated to be 616 million (Carapetis et al., 2005), while it has been speculated that there are 162 million children affected by impetigo at any one time (Bowen et al., 2015). GAS also causes sequelae, including rheumatic heart disease and acute glomerulonephritis, as well as streptococcal toxic-shock syndrome. The major typing scheme involves M and T serotyping. The former is based on the antigenicity of the M protein encoded by the *emm* gene. The 90 bp DNA sequence encoding the N-terminal variable region of the mature M protein is utilized to classify GAS into more than 240 types, known as *emm* typing (Beall et al., 1996; Sanderson-Smith et al., 2014). T typing is an alternative scheme based on the antigenicity of trypsin-resistant antigens (T antigens) (Griffith, 1934; Lancefield, 1940; Lancefield and Dole, 1946). It is conducted using trypsin-treated GAS cells and hyperimmune rabbit typing serum. The typing serum is raised against trypsin-treated GAS surface proteins, followed by adsorption with undigested GAS cells of different T types. Commercial antisera consists of five types of polyvalent sera and 21 of monovalent sera (Takizawa et al., 1970). A drawback of T serotyping is a lack of specificity. Several different typing sera react with recombinant pilus tip minor subunits (Lizano et al., 2007; Falugi et al., 2008; Nakata et al., 2009). Also, GAS isolates often react with several typing sera, such as T3/13/B3264 (Falugi et al., 2008). Thus, there is a lack of resolution compared to M typing. Moreover, unlike *emm* typing, there is no widespread genotyping method for pilus genes or the FCT region. Mora et al. (2005) reported that the major pilin of the GAS pilus is responsible for the antigenicity of T typing. The mechanism of protease resistance of a major pilin T antigen was uncovered by solving the structure by X-ray crystallography (Kang et al., 2007).

Over the last decade GAS pili have been found to be responsible for several functions, including host cell adherence, biofilm formation, immune evasion, and virulence. In this review, advances in understanding of pilus functions, the mode of pilus expression, perspectives for pilus-based vaccines, and the application of the structure and mechanisms of assembly of pili to biotechnology are summarized.

## Genetic Organization of FCT Genomic Region Containing Pilus-Related Genes

**FIGURE 1:**
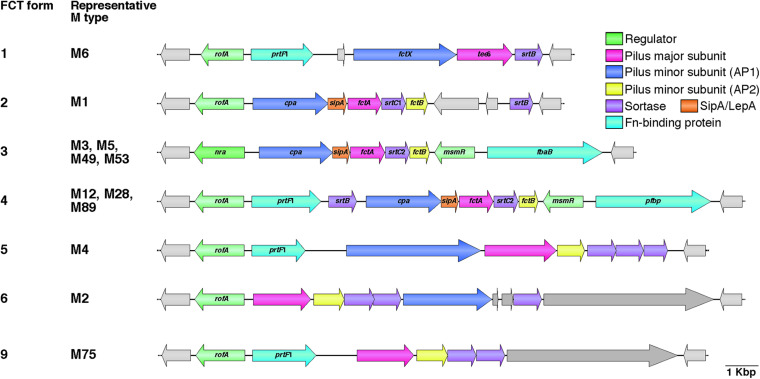
Heterogeneic organization of FCT region. Gene content heterogeneity for the seven FCT forms is shown on the basis of genome sequences and previously reported data (Kratovac et al., 2007; Falugi et al., 2008). Representative M types for each FCT form are also shown. Pilus major and minor subunit (ancillary proteins 1 and 2; AP1 and AP2) genes are shown in pink and blue, respectively. SipA/LepA and sortase genes are colored orange and purple, respectively. Fibronectin-binding protein genes, including *prtF*2 family genes (*pfbp* and *fbaB*) and *prtF*1, are shown in green, while transcriptional regulator genes, including *rofA*, *nra*, and *msmR*, are shown in light green. Other genes are light gray in color. Deposited DNA sequences of strains (M type, accession number) used for each FCT form are as follows: form 1, MGAS10394 (M6, NC_006086); form 2, SF370 (M1, NC_002737); form 3, SSI-1 (M3, NC_004606); form 4, A735 (M12, AF447492); form 5, MGAS10750 (M4, NC_008024); form 6, MGAS10270 (M2, NC_008022); and form 9, STAB14018 (M75, CCP014542.1). Genome sequences for FCT forms 7 and 8 are not available.

The genes that specify pili are located in the FCT genomic region (Bessen and Kalia, 2002). The acronym FCT stands for fibronectin-binding proteins, collagen-binding proteins, and trypsin-resistant antigens. Prior to discovery of pili, the gene encoding the T antigen type 6 (*tee*6) was reported (Schneewind et al., 1990). A BlastN search with *tee*6 and the flanking sequences as the query revealed a short stretch of sequence identity (91% over 70 bp) between the downstream region of *tee*6 and intergenic regions of *spy*0133 and *spy*0135 in the M1 strain SF370. Subsequent comparative analyses of deposited DNA sequences from additional serotypes revealed an approximately 11–16 kb recombinatorial region (Bessen and Kalia, 2002). This region contains genes encoding the fibronectin-binding F1/SfbI (Sela et al., 1993; Talay et al., 1994) and F2/PFBP/FbaB (Jaffe et al., 1996; Rocha and Fischetti, 1999; Terao et al., 2002) proteins, as well as the collagen-binding Cpa protein (Kreikemeyer et al., 2005). The FCT region is located between the conserved genes *hsp*33 and *spy*0136 (genome of SF370), and positioned nearly equidistant from the replication origin (clockwise from *ori*) as the M protein-coding *emm* locus. The heterogeneity of gene content among different GAS strains has allowed classification into nine subtypes designated FCT forms 1–9 (Figure 1, hereafter referred to as FCT-1 to FCT-9) (Kratovac et al., 2007; Falugi et al., 2008). To the best of our knowledge, the genome sequences of all except FCT-7 and FCT-8 are available. Generally, the same *emm* type strains share the same FCT form, though some exceptions have been reported (Falugi et al., 2008; Köller et al., 2010; Steemson et al., 2014). This region also encodes three kinds of transcriptional regulators, namely Nra, RofA, and MsmR (Fogg et al., 1994; Podbielski et al., 1999; Nakata et al., 2005). RofA and Nra belong to the RofA-like protein (RALP) regulator family that consists of four members with a mean amino acid sequence identity of 29% (Granok et al., 2000). Among these three regulators, Nra and RofA show an approximately 62% protein identity. The *nra* gene occurs in FCT-3, while other FCT forms contain the *rofA* gene. MsmR, an AraC-type regulator, is specific to FCT-3 and -4, and is always encoded by a gene located adjacent to *prtF*2 family genes (Nakata et al., 2005).

Pilus-related genes constitute an operon and encode one major and one or two minor subunits, at least one pilin-specific SrtB or SrtC type sortase, and the FCT-form specific chaperone SipA/LepA (Barnett and Scott, 2002; Barnett et al., 2004; Zähner and Scott, 2008). Based on primary amino acid sequences, five sortase classes have been defined as SrtA to SrtF (Dramsi et al., 2005; Spirig et al., 2011). Class B sortases are predominant in Firmicutes, and their functions include pilus assembly and cell wall anchoring of proteins involved in iron acquisition (Mazmanian et al., 1999; Mora et al., 2005). Class C sortases are predominant in Firmicutes and Actinobacteria, and specifically function in pilus assembly. Confusingly, the GAS SrtB and SrtC proteins belong to class C and B, respectively, and both function in pilus assembly (Kang et al., 2011; Nakata et al., 2011). The major and minor subunits are often denoted as the backbone protein (bp) and ancillary proteins (ap), respectively. The number of minor ap subunits varies among types, with the tip minor subunit and base subunit usually termed ap1 and ap2, respectively. Falugi et al. analyzed seven different FCT forms and showed that the major subunit bp can be grouped into 15 variants. This was later expanded to 18 variants. There are 14 and 5 variants for the minor subunits ap1 and ap2, respectively (Falugi et al., 2008; Steemson et al., 2014). They also demonstrated that the major subunit bp is mainly responsible for T serotyping specificity.

Among the FCT forms, FCT-3 and FCT-4 share the greatest similarity. Inter-strain recombination of pilus genes between FCT-3 and FCT-4 has been speculated based on phylogenetic analysis and findings showing that an M5 strain possessed a *cpa* gene from FCT-4 (Falugi et al., 2008). Interestingly, the FCT-6 pilus minor subunit genes of several M2 strains show considerable homology to Group B *Streptococcus* pilus island I (PI-1) minor subunit genes, while the FCT region (FCT-1) of the M6 strain D471 has homology with the *rlrA* pathogenicity islet of *S. pneumoniae* (Bessen and Kalia, 2002; Hava and Camilli, 2002; Barocchi et al., 2006; Falugi et al., 2008). Horizontal gene transfer and recombination seems to have occurred between related species.

While *emm* typing is based on the sequence of 5′-end of the *emm* gene, the 3’-ends of *emm* and *emm*-like genes encoding M-like proteins, such as Mrp and Enn (Frost et al., 2018; Frost et al., 2020), are used for *emm* pattern groupings (Bessen et al., 1996). A strong correspondence between three groupings (patterns A-C, D, and E) and infection site preference, i.e., throat or skin, has been shown. Based on epidemiological data, *emm* pattern A-C and pattern D strains are designated “throat specialists” and “skin specialists,” respectively, while pattern E strains are designated “generalists.” The correlation between *emm* and FCT forms has been emphasized by data showing that 83% of FCT-3 strains harbor *emm* pattern D, whereas 84% of FCT-4 strains harbor *emm* pattern E (Kratovac et al., 2007). This strong linkage between FCT form and *emm* pattern raises the possibility that factors encoded in the FCT region, including pili, have roles in tissue tropism.

## Assembly of Gas Pili

**FIGURE 2:**
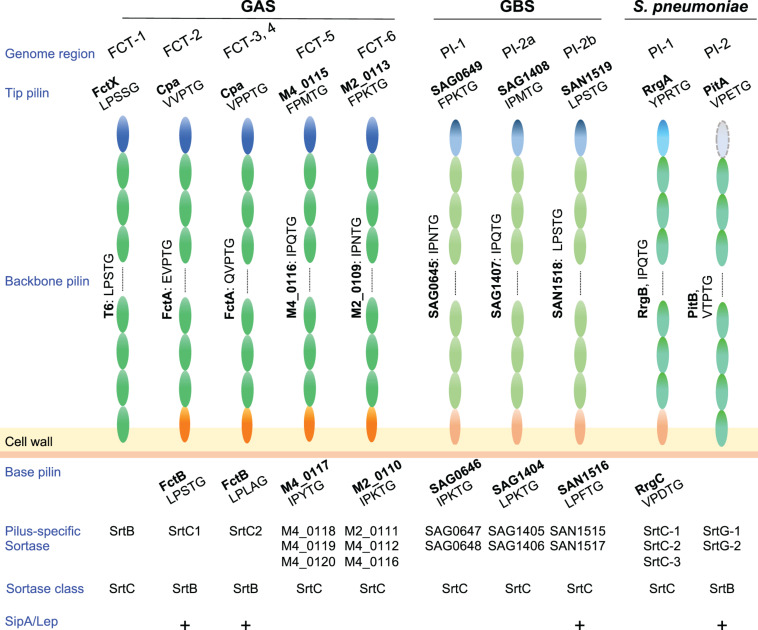
Pilus components and related factors of pathogenic streptococci. Pilus major (backbone pilin) and minor (tip and base pilin) subunits of *Streptococcus pyogenes* (GAS), *Streptococcus agalactiae* (GBS), and *Streptococcus pneumoniae* are depicted. GBS pilus genes are located in three pilus island (PI) types, while *S. pneumoniae* pilus genes are located in two types of pilus islets (PIs). LPXTG or an LPXTG-like motif is shown under each pilin. Related pilus-specific sortases and their class, i.e., sortase class B or C, are also shown. Note that GAS SrtB and SrtC1/2 belongs to class C and B, respectively. A requirement of SipA/LepA for pilus assembly is also shown by “+.” Pilin subunits of FCT forms 5 and 6 are presented as gene tag numbers of MGANC_008024S10750 (serotype M4, genome accession number NC_008024) and MGAS10270 (serotype M2, genome accession number NC_008022), respectively. Subunits of GBS pili are also shown as tag numbers in 2603V/R (serotype V, genome accession number NC_004116.1) and COH1 (serotype III, genome accession number HG939456).

In Gram-positive bacteria pilus subunits are linked to each other by isopeptide bonds mediated by pilus-specific sortases encoded in pilus gene clusters (Hendrickx et al., 2011). Among pilus types of pathogenic streptococci, the number of pilus-specific sortases varies (Figure 2). In GAS, FCT-5 and FCT-6 strains contain multiple class C sortases, as observed for pili of *S. agalactiae* and *S. pneumoniae*, while there is only one class B or class C sortase in FCT-1 to FCT-4. Pilin subunits possess a secretory signal sequence at their N-termini and a C-terminal cell wall sorting signal (CWSS) containing an LPXTG or LPXTG-like motif. This is followed by a stretch of hydrophobic residues and a positive-charged anchor that retains subunit proteins in the membrane during secretion via the Sec apparatus. The pilus-specific sortase cleaves an LPXTG or LPXTG-like motif between the threonine and glycine residues, and subsequently forms an acyl-enzyme intermediate by linking the active cysteine residue to the carboxyl group of the threonine. This intermediate is relieved by nucleophilic attack by the lysine residue side chain in the adjacent pilus subunit, forming isopeptide bonds between adjacent subunits. A series of consecutive reactions elongate pili until the occurrence of a stop signal, namely the incorporation of a minor subunit containing the canonical LPXTG motif (Smith et al., 2010), allowing the assembled pili to be connected to a free amino group of the peptidoglycan layer by the housekeeping sortase SrtA (Mazmanian et al., 1999. Thus far, assembly mechanisms have been mainly investigated for FCT-1, FCT-2, and FCT-3 pili in GAS.

Among the nine FCT forms, FCT-2, FCT-3, and FCT-4 pili comprise three components, including the major subunit FctA, and minor subunits Cpa (ap1) and FctB (ap2) (Figure 2). The pilus-specific sortase has been named SrtC. It comprises two alleles; SrtC1 in FCT-2 and SrtC2 in FCT-3 and FCT-4 (Barnett et al., 2004; Dramsi et al., 2005; Spirig et al., 2011). In an M3 strain, SrtC2 was shown to be responsible both for linkage between Cpa and FctA, as well as FctA polymerization (Quigley et al., 2009). FctA and Cpa in FCT-3 contain LPXTG-like VPPTG and QVPTG sorting sequences, respectively, with isopeptide bonds formed with K173 of FctA (Quigley et al., 2009). Those authors also reported that Cpa is located exclusively at the pilus tip. The corresponding LPXTG-like sequences of Cpa and FctA in M1 strains (FCT-2) are EVPTG and VVPTG, respectively, with the slight differences in substrate sequences likely attributed to variations in the substrate recognition of two SrtC alleles and two SipA/LepA alleles (Figure 2). Covalent linkage between the C-terminal threonine of Cpa and a lysine residue of FctA was also reported in an M1 strain (Smith et al., 2010). For anchoring of pili to the cell wall by SrtA, that report also noted that the minor subunit FctB was incorporated into the base of the Cpa-FctA complex as a stop signal for FctA polymerization. The FctB protein of M1 strains contains a canonical LPXTG motif (LPLAGE in FCT-3), which is a substrate for SrtA. A distinctive feature of FCT-2, FTC-3, and FCT-4 pili is that their assembly requires the signal peptidase I homolog SipA/LepA, the gene for which is also located in the pilus gene operon (Zähner and Scott, 2008; Nakata et al., 2009). Catalytic residues are not conserved in SipA/LepA, and *in vitro* assays using peptide fragments of pili and recombinant SipA/LepA show no peptide cleavage. Thus, SipA/lepA has been suggested to act as a molecular chaperone that coordinates pilus assembly with SrtC (Young et al., 2014b). The signal peptidase I homologs in *S. agalactiae* and *S. pneumoniae* are also required for assembly of pili (Figure 2; Bagnoli et al., 2008; Périchon et al., 2019), the genes for which are situated in pilus island II b (PI-2b) and the pathogenicity islet 2 (PI-2), respectively.

Assembly of FCT-1 pili in an M6 strain has been investigated (Nakata et al., 2011). Pili are composed of the major T6 subunit and minor FctX subunit as a tip protein. The CWSSs of T6 and FctX include an LPSTG and LPSSG sequence, respectively (Schneewind et al., 1990; Bessen and Kalia, 2002). The K175 residue of T6 was shown to participate in T6 polymerization as well as linkage of T6 and FctX (Nakata et al., 2011; Young et al., 2014a). The pilus-specific sortase SrtB belongs to the SrtC family and is primarily required for efficient pilus assembly while SrtA is responsible for cell wall anchoring of T6 pili (Nakata et al., 2011). Since deletion of the *srtB* gene does not completely abrogate T6 polymerization or formation of an FctX-T6 complex, as shown by immunoblot assay results, it is likely that SrtA can compensate for the loss of SrtB in pilus assembly to a certain extent (Nakata et al., 2011). Unlike FCT-2 and FCT-3 pili, there is no minor subunit for a stop signal and the mechanism of stopping polymerization remains unknown. Furthermore, several studies have demonstrated that deletion of the gene encoding the pilus tip protein (ap1) decreases the detection level of polymerized major subunits in FCT-1, FCT-2, FCT-3, and FCT-6 pili. This prompted speculation that heterodimer formation between the major and minor tip subunits accelerates polymerization of the major subunits (Lizano et al., 2007; Nakata et al., 2009, 2011; Tsai et al., 2017).

## Regulation of Pilus Gene Expression

GAS pilus-related gene expression has been shown to be mediated by RALP family transcriptional regulators, including RofA and Nra, in a serotype- or strain-dependent manner. Previous reports have indicated that both RofA and Nra can function as autoregulators (Podbielski et al., 1999; Granok et al., 2000), and expression of pilus genes in FCT-3 strains is positively or negatively regulated by Nra in a strain-specific manner (Podbielski et al., 1999; Luo et al., 2008). Recent studies of in-frame deletion mutants indicated that Nra acts as a positive regulator in several M3 strains and an M49 strain (Calfee et al., 2018; Nakata et al., 2020). RofA has been reported to be a positive regulator for the protein F1 gene (*prtF*1) (Fogg et al., 1994). Also, the involvement of RofA in pilus gene expression was indirectly shown by replacement of *nra* with *rofA* along with respective upstream promoter regions from an M6 strain in the background of an M53 strain (FCT-3) (Lizano et al., 2008). The replacement resulted in preserved pilus gene expression. On the other hand, deletion of the *rofA* gene reduced pilus gene expression in an M1 strain (Calfee et al., 2018). Thus, RALP family members, such as Nra and RofA, likely promote pilus gene expression. In addition, MsmR, Mga, and RALP3 can influence pilus gene expression in a strain-specific manner (Nakata et al., 2005; Kreikemeyer et al., 2007; Kwinn et al., 2007; Luo et al., 2008).

*In vitro* induction of pilus gene expression occurs under a variety of culture conditions including low pH and low temperature (Nakata et al., 2009; Manetti et al., 2010). A topic gaining increasing focus is the molecular mechanisms that underlie modulation of pilus gene expression in response to environmental signals, such as temperature and acidity. The level of pilin detection is altered by shifting the culture temperature. Utilizing an M49 strain, we showed increased FctA expression at 30°C as compared to 37°C. Moreover, the expression of FctA appeared to be bistable, as only some cells in *S. pyogenes* chains were FctA-positive in immunofluorescence experiments (Nakata et al., 2009). Furthermore, decreasing the temperature to 25°C induced pilus production by the majority of cells (Nakata et al., 2020). This bistabilty was later characterized in more detail for type 1 pilus genes from pneumococci and shown to depend on the positive regulator RlrA acting in a positive feedback loop on pilus genes (Basset et al., 2011, 2017). Moreover, such bistabilty has clear implications for infections, as pilus-1 was shown to be preferentially expressed during early colonization in animal infection models (Pancotto et al., 2013). It remains to be determined if bistability of *S. pyogenes* pilus expression has any implications for *in vivo* pathogenesis ecology.

**FIGURE 3:**
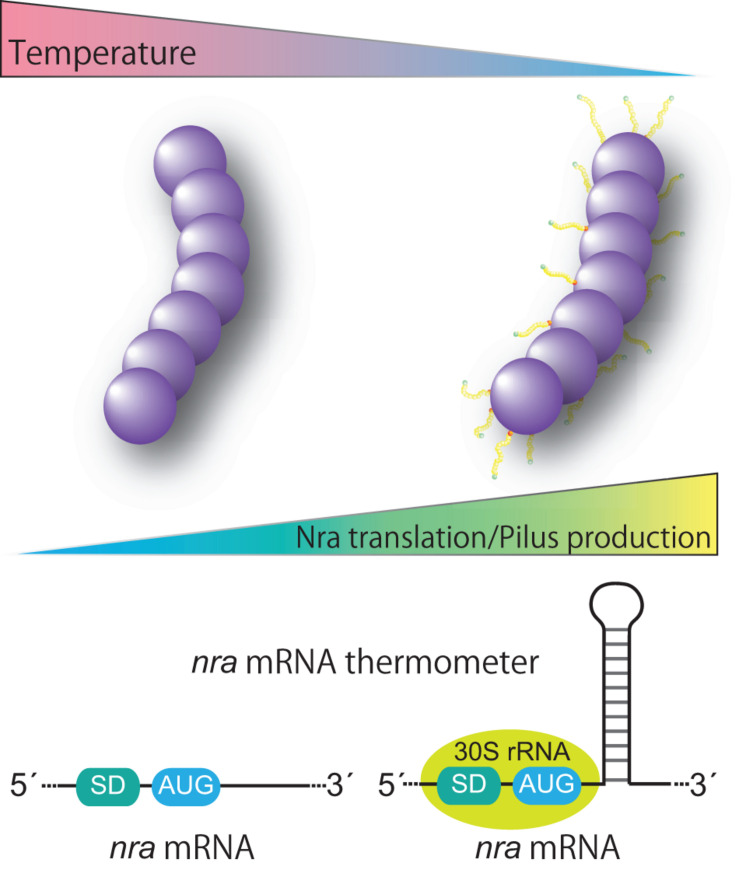
Proposed model for thermoregulation of pilus production from FCT form 3 strains. FCT form 3 *nra*-positive *Streptococcus pyogenes* produces pili in a temperature-dependent manner. The underlying mechanism includes post-transcriptional control of *nra* mRNA translation via a putative stem loop structure in the protein coding region of *nra* mRNA. The putative stem loop structure most likely functions as a thermometer to modulate the translational efficiency of *nra* mRNA by potential interactions with the translation initiation complex. Thermosensitive modulation of pilus production highlights the importance of pili in an initial infection phase and involvement of pili in bacterial fitness in the host.

Historically, GAS cultures for a T-typing test have been grown at 30°C (Griffith, 1934). Thermosensitive pilus production occurs at the transcriptional level and the expression pattern is restricted to *nra*-positive FCT-3 strains (Nakata et al., 2009, 2020). The underlying mechanism involves post-transcriptional control of *nra* mRNA translation, namely promoting translation at low temperatures (Figure 3). Of note, in a study that utilized an M3 and an M49 strain, introduction of silent base substitutions in the chromosome to melt the predicted stem loop structure located 23 bases downstream of the AUG start codon decreased detection of both the Nra protein and pili especially at low temperatures (Nakata et al., 2020). Considering that temperature at the initial infection site is lower than the core body temperature and formation of mRNA stem-loop structure is influenced by temperature, we speculated that the predicted stem-loop structure is an mRNA thermometer within *nra* mRNA. It might form the stable base-pairing at lower temperatures, and could be more susceptible to melting at the core body temperature. Thus, lower temperatures reflecting the initial infection site would promote the *nra* translation and subsequent pilus gene expression, thereby promoting colonization. If temperature increased due to bacterial invasion of tissues and inflammation, pilus production by FCT-3 strains would be halted. How the stem loop promotes translation of *nra* mRNA remains elusive. It possibly involves a “starting block” mechanism, whereby the stem-loop prevents the 30S ribosomal subunit from sliding onto mRNA, optimizing the positioning of 30S rRNA and promoting translation initiation (Figure 3; Jagodnik et al., 2017). Although additional experimental confirmation is required to elucidate the mechanism, the existence of a temperature-perception system in the pilus gene transcriptional regulator adds a new level of regulation to virulence factor expression in GAS. Furthermore, Kratovac et al. noted that among 39 *emm* types associated with FCT-3, 32 (88.8%) represented pattern D of skin specialists (Kratovac et al., 2007; Bessen, 2016) raising the possibility of a link between thermosensitive pilus expression of FCT-3 strains and skin tropism. The molecular interactions between pili and host factors in skin have yet to be elucidated. Moreover, whether regulation of pilus gene expression studied *in vitro* matches the *in vivo* situation and thereby could be translated into clinical scenarios remains to be investigated. If pilus expression *per se* contributes to switching *S. pyogenes* lifestyles, it will most likely occur in a serotype- or even strain/isolate-specific manner.

Upregulation of pilus expression by acid stress is also conducted by two-component systems. Comparisons of genome sequences of M3 (FCT-3) isolates recovered from symptomatic pharyngitis and subsequent asymptomatic carriage in the same patient (at day 63 post infection) revealed three single nucleotide polymorphisms, including one mutation in the sensor kinase LiaS (R135G) of the LiaFSR three-component system (or YvqE of the YvqEC two-component system) (Flores et al., 2015). The mutation was shown to alter the transcriptome. It also resulted in an increased ability to adhere to cultured epithelial cells and to colonize nasopharynx tissues, increased susceptibility to antibiotics targeting cell wall synthesis and decreased virulence in a mouse model of necrotizing fasciitis (Flores et al., 2015). An M3 strain with the R135G substitution in LiaS expressed pili at a higher level when exposed to bacitracin, a condition which is known to promote *liaFSR* expression. It seems that bacitracin did not promote the pilus gene expression in the R135G mutant (Flores et al., 2017). On the other hand, LiaS was demonstrated to play an important role in virulence and to sense acidic conditions, as an *liaS* mutant of an M1 strain had reduced growth at pH 6.0 (Ichikawa et al., 2011). Also, an *liaS* mutant produced less acid during sugar fermentation (Isaka et al., 2016). LiaS also controls pilus production and biofilm formation under acidic conditions. Introduction of the D26N substitution into a predicted extracellular region of LiaS compromised acid production and biofilm formation. This indicates that sensing organic acid by the LiaS extracellular domain is connected to the relationship between pilus expression and pH (Manetti et al., 2010; Isaka et al., 2016). It is not known if LiaS directly regulates pilus gene expression. Deletion of the homologue of *liaS* in *S*. *pneumoniae* and *S*. *agalactiae* also showed altered pilus expression (Rosch et al., 2008; Klinzing et al., 2013).

Non-coding RNA (ncRNA) is a crucial element in modulating virulence factor expression (Caldelari et al., 2013). Genome-wide tiling array and differential RNA sequencing analyses of GAS ncRNAs together with bioinformatic and expression analyses revealed a *bona fide* expression of ncRNA in M1, M3, and M49 strains, including crRNA and trans-activating RNA of the Cas9-CRISPR system (Perez et al., 2009; Raasch et al., 2010; Deltcheva et al., 2011; Patenge et al., 2012, 2015; Tesorero et al., 2013). Among GAS ncRNA *fasX* negatively regulates translation of *cpa* in an M1 strain, *tee*6 in an M6 strain, the gene encoding a minor subunit (ap1) in an M2 strain, and *fctA* in an M28 strain (Liu et al., 2012; Danger et al., 2015). The mechanism involves base pairing of *fasX* with ribosome-binding sites, which leads to a reduction in mRNA stability and translation. *fasX* ncRNA is under the control of the FasBCA two-component system, originally reported to promote streptokinase production and haemolysis, and downregulating fibronectin/fibrinogen binding (Kreikemeyer et al., 2001). The environmental cues leading to expression of *fasBCAX* remain unknown. The levels of *fasX* expression show intraspecies variability as well as differences among FCT-3-associated serotypes including M3. For example, many M3 strains harbor a 4 bp deletion in the *fasC* gene encoding a histidine sensor kinase and consequently *fasX* expression is relatively low, which promotes pilus expression (Perez et al., 2009). However, inter-serotype transcriptome results demonstrated that several M3 strains have lower amounts of pili compared to several M1 and M49 strains due to a lower level of *nra* expression (Calfee et al., 2018). Together with frameshift mutations in *rocA* and *rivR* encoding a pseudo-kinase and transcriptional regulator, respectively (Biswas and Scott, 2003; Roberts et al., 2007), the loss of *fasX* regulation contributes to a selective advantage for M3 strains (Sarkar and Sumby, 2017).

The CovRS (or CsrRS) system is one of the most studied two-component systems and known to regulate approximately 15% of GAS genes (Graham et al., 2002). A prominent feature of this system is downregulation of several different virulence factors and natural mutations of these genes are strongly correlated with the onset of invasive diseases. A mutation of *covR* encoding the response regulator promoted pilus expression in several FCT-3 strains of serotypes M3 and M49, but not in an FCT-2 M1 strain (Kreth et al., 2011; Horstmann et al., 2015; Calfee et al., 2018) reflecting the fact that the intergenic regions of *nra-cpa* and *rofA-cpa* are divergent. Thus, counteractive modulation of pilus expression is governed by Nra and CovRS in large numbers of FCT-3 strains.

As shown by these findings, regulation of pilus expression is governed by various factors, including transcriptional regulators, ncRNA, and the mRNA thermometer. An intricate interplay among those factors shapes expression in response to the extracellular milieu and intracellular metabolic activity of the infected host cell. The FCT form exhibiting specific patterns of pilus expression highlights the importance of pili in regard to adaptation to host environments and their requirement for causing a variety of diseases. Further elucidation of these regulatory mechanisms is warranted.

## Biological Functions of Gas Pili

The various functions of the GAS pili are summarized in Table 1.

**TABLE 1:** Involvement of GAS pili in host cell adherence, interactions with host molecules, biofilm formation, and virulence.

FCT form	M type (T type)	Host cell ADHERENCE Cell lines/tissues—adhesin	Interactions between host molecules and pilin	Involvement in biofilm formation*	Effects of pilus gene deletion on virulence in infection models (Host)*
FCT-1	M6 (T6)	A549—FctX	Gp340—FctA	+	Decreased virulence I. p. infection (CD1 mouse)
FCT-2	M1 (T1)	HaCaT, Detroit-562—Cpa Tonsil epithelium	Gp340—FctA	+	Increased virulence S. c. infection (CD1 mouse)
FCT-3	M3 (T3) M49 M53	HaCaT	Human collagen type I—Cpa (M49) Gp340—FctA (M3)	– (M49)	Decreased virulence S. c. infection (Human skin-engrafted SCID mouse) (M53)
FCT-5	M4 (T4)	HaCaT, RPMI 2650	Haptoglobin—SPyM4_0116^*a*^	+	Decreased virulence S. c. and i.p. infection (CD1 mouse)
FCT-6	M2 (T2)	HaCaT, Detroit-562 - SPyM2_0109^#^	Fibronectin and Fibrinogen—SPyM2_0109^#^		Decreased virulence Infection into the lower left proleg (*Galleria mellonella*)

*I. p., intraperitoneal; S. c., subcutaneous.^a^Tag number of MGAS10750 (genome accession number, NC_008024).^#^Tag number of MGAS10270 (genome accession number, NC_008022).

### Adhesion to Host Cells

A diverse array of secreted and surface-anchored components can mediate host cell adherence. These include cell wall anchored surface proteins, namely MSCRAMMs (microbial surface components recognizing adhesive matrix molecules, Patti et al., 1994), such as M proteins and a variety of fibronectin-binding, laminin-binding, and collagen-binding proteins. Other surface components are lipoteichoic acid, a hyaluronic acid capsule, and moonlighting proteins, including streptococcal enolase and glyceraldehyde-3-phosphate dehydrogenase (Brouwer et al., 2016). Cultured cell lines such as HEp-2 (HeLa derivative) and A549 (human alveolar adenocarcinoma cell line) have been frequently used to identify and analyze those factors. Abbot et al. (2007) utilized both cell lines and showed that an *fctA* deletion in the M1 strain SF370 had no effect on bacterial adherence. On the other hand, Crotty Alexander et al. reported that an *fctA* deletion in M1 strain 5448 induced a slight though statistically significant decrease in bacterial adherence to HEp-2 cells, although complementation failed to recover completely the reduction in adherence (Crotty Alexander et al., 2010). Importantly, T1 pili promoted adhesion to clinically relevant tissues in the throat and skin. Abbot et al. (2007) clearly demonstrated T1 pili binding to freshly isolated human tonsil tissues and to primary human keratinocytes, as well as to the human keratinocyte cell line HaCaT. Adhesion of M1 strain SF370 to the pharyngeal cell line Detroit 562 also showed T1 pili dependence (Manetti et al., 2007; Smith et al., 2010).

The above-mentioned findings raised questions regarding how T1 pili recognize host cells and which pilin component is responsible for binding. Flow cytometric analyses with recombinant pilin components revealed that recombinant Cpa and FctB, but not FctA, bound to the surface of Detroit 562 cells (Manetti et al., 2007). However, inhibition assays with antisera against each pilus subunit indicated that only anti-Cpa1 serum significantly inhibited bacterial adherence to both HaCaT cells and human tonsil epithelium (Smith et al., 2010). The central region of Cpa1 extending from Asn286 to Pro559 was responsible for bacterial adhesion via pili. Indeed, Cpa in T9 pili was considered to be a molecular harpoon that exerts adhesion via its own amine-reactive thioester bonds (Linke-Winnebeck et al., 2014), suggesting that Cpa plays a central role in cell recognition. It is likely that FctA polymerization allows Cpa to be located away from the cell surface and placed in the vicinity of host cells. This scheme would also be applicable to *cpa* operon-positive FCT-3 and FCT-4 forms. Involvement of either a major or minor subunit of other FCT-form pili in host cell adherence has also been reported. Regarding FCT-1 pili, the minor subunit FctX was shown to contribute to adherence of an M6 strain to A549 cells (Becherelli et al., 2012). In contrast, the tip pilin protein (SPyM2_0113) of an M2 strain (FCT-6) did not promote adhesion to HaCaT or Detroit-562 cells (Tsai et al., 2017). Instead, the major subunit T2 (SPyM2_0109) promoted adherence. Thus, in general, GAS pili can function as primary adhesins during the initial stages of colonization in the upper respiratory tract or skin.

Details regarding pilus receptors remain elusive, though some interactions between tip pilin and host molecules have been reported. The tip pilin Cpa from an M49 strain binds human collagen type I (Kreikemeyer et al., 2005). A high affinity interaction with a Kd in the nanomolar to low micromolar range was measured by ELISA-type assays and surface plasmon resonance (Kreikemeyer et al., 2005). This somehow resembles function of the collagen adhesion Cna of *Staphylococcus aures*. Interestingly, binding of major pilus subunits to host proteins has also been reported. FCT-1, FCT-2, and FCT-3 pili bind the salivary glycoprotein gp340 and the major subunit T2 binds fibronectin and fibrinogen (Edwards et al., 2008; Tsai et al., 2017). FctA plays a major role in interactions between FCT-2 T1 pili, but not FCT-4, and gp340. Binding of gp340 to bacteria mediates bacterial aggregation and it also inhibits bacterial adhesion to Detroit-562 and HeLa cells. Since gp340 binds to secretory IgA and complement C1q (Madsen et al., 2010) bacterial aggregation via gp340 binding may promote bacterial clearance and contribute to innate immunity.

### Bacterial Aggregation and Biofilm Formation

Similar to many other pathogenic bacteria, GAS forms micro-colonies and biofilm on both biotic and abiotic surfaces. These actvities have been shown in numerous *in vitro* studies as well as *in vivo* infection models with various hosts, including mice, zebrafish, and chinchillas (Neely et al., 2002; Roberts et al., 2010; Connolly et al., 2011). More importantly, micro-colonies and biofilm-like structures have been found in clinical specimens of human impetigo lesions and tonsil tissues (Akiyama et al., 2003; Roberts et al., 2012). The 3-D structure of bacterial biofilm is defined by sessile cells being encased in a matrix of extracellular polymeric substances comprising proteins, DNA, and a glycocalyx. Biofilm-embedded bacteria exhibit a low growth rate and reduced metabolism, which poses problems when attempting antibiotic therapy (Donlan and Costerton, 2002). Moreover, several clinical studies have noted that the ability of GAS to form biofilm is related to recurrent infection episodes (Roberts et al., 2010; Torretta et al., 2012). Therefore, GAS biofilms are likely to be clinically relevant and therapeutic approaches against them may be effective for infection control. The ability of GAS strains to form biofilm varies and *in vitro* conditions required for biofilm development differ among strains. A coating of matrix or serum proteins can promote biofilm formation on an abiotic surface (Lembke et al., 2006) so adhesins such as MSCRAMMS likely contribute to biofilm formation.

A systemic evaluation of relationships between biofilm formation and FCT forms was conducted using 183 clinical isolates in Germany (Köller et al., 2010). This study showed that all tested FCT-1 strains, including M6 strains, efficiently formed massive biofilms in peptide-rich but carbohydrate-poor C medium, as well as in enrichment medium such as brain heart infusion (BHI) broth. Several FCT-2, FCT-5, and FCT-6 strains also formed biofilms in BHI, though to a lesser extent in C-medium, while two FCT-9 strains formed weak biofilms regardless of the culture medium. Furthermore, FCT-3 and FCT-4 strains showed a widely varying ability to form biofilm in both types of media. These phenotypic variations indicate that the ability to form biofilm is affected, at least in part, by culture conditions and can be roughly grouped by FCT form, although strain specificity occurs within some FCT forms. Several studies have shown a direct role for pili in biofilm formation (Manetti et al., 2007; Becherelli et al., 2012; Kimura et al., 2012; Chen et al., 2020). In the study of Manetti et al., T1 pili promoted *in vitro* biofilm formation by an M1 strain on polylysine-coated glass via aggregation and microcolony formation. On the other hand, biofilm formation of a M49 strain (FCT-3) was not affected by several mutations of pilus-related genes (Nakata et al., 2009). The remarkable ability of M6 strains to form biofilm was shown to be attributable to T6 pilus production (Kimura et al., 2012). Deletion of the gene encoding either the major (T6) or minor (FctX) subunit decreased biofilm formation, while the same was true when the *srtB* gene encoding the pilus-specific sortase was deleted. Surprisingly, as compared with the parental strain, these mutant showed increased bacterial aggregation. When the entire group of T6 pilus-related genes was ectopically expressed in the M1 strain SF370, biofilm formation was promoted and aggregation inhibited (Kimura et al., 2012). It seems that T1 and T6 pili mediate biofilm formation by different mechanisms. FctA pili mediate biofilm formation by auto-aggregation and microcolony formation, while the T6 pilus functions as an adhesin responsible for initial attachment leading to biofilm formation. However, the contribution of pili to aggregation remains controversial. Becherelli et al. (2012) reported that FctX exhibited homophilic interactions that mediated inter-bacterial contact, thereby mediating aggregation. The phenotypic difference in aggregation of the *fctX* mutant strain could be attributable to differences in experimental conditions. Further analysis is required before drawing a final conclusion. Becherelli et al. (2012) also reported that homophilic interactions were observed for Cpa with serotypes M1 and M3 strains, suggesting a general mechanism of aggregation via homophilic interactions between minor subunits.

Among culture conditions examined, an acidic environment facilitates biofilm formation by specific FCT form strains (Manetti et al., 2010). The authors compared the biofilm forming ability in C-medium at pH 6.4 and 7.5, and found that the lower pH condition was favorable for biofilm development with strains from FCT-2 (M1), FCT-3 (M3 and M5), FCT-5 (M4), and FCT-6 (M2). Pilus production was also upregulated at the lower pH, indicating a pH-dependent relationship between biofilm formation and pilus production. On the other hand, pH levels did not influence the biofilm forming ability of M28 and M89 strains belonging to FCT-4 or M75 strains belonging to FCT-9, though biofilm mass was relatively low. FCT-1 strains (M6 and M109) efficiently formed biofilms under both conditions. Thus, strains with specific FCT forms have the ability to sense the environmental acidity and form biofilm via increased pilus production. Such differential response to environmental signals influences variations in biofilm formation. For more details regarding GAS factors involved in biofilm formation please refer to the review of Fiedler et al. (2015).

### Virulence in Infection Models

The relationship between GAS pilin expression and virulence has been examined in strains belonging to FCT-1, FTC-2, FCT-3, FCT-5, and FCT-6. The extent to which each pilus type promotes or attenuates virulence varies, indicating differences among the forms (Lizano et al., 2007; Luo et al., 2008; Nakata et al., 2009; Crotty Alexander et al., 2010; Becherelli et al., 2012; Rouchon et al., 2017; Tsai et al., 2017; Chen et al., 2020). The contribution of FCT-1 T6 pili to pathogenesis was examined in a mouse intraperitoneal infection model, which indicated that the minor subunit FctX contributes to bacterial dissemination to the spleen, lungs, and kidneys, as well as survival in blood (Becherelli et al., 2012). In contrast, FCT-2 T1 pili reduced virulence in a murine subcutaneous infection model and decreased bacterial survival in human blood. Additionally, FCT-2 T1 pili had no influence on neutrophil phagocytosis, complement deposition in human sera, or sensitivity to the cathelicidin-related antimicrobial peptide LL-37. However, FCT-2 T1 pili induced neutrophil IL-8 production, neutrophil endothelial transcytosis, and neutrophil extracellular traps (NETs), thereby promoting entrapment and killing of GAS via NETs. Utilizing a human skin-engrafted SCID mouse line and an M53 skin-tropic strain (FCT-3), Lizano et al. evaluated the role of the Cpa and FctA pilus subunits in superficial skin infection. Deletion of the gene encoding Cpa attenuated virulence, while the *fctA* mutant showed virulence comparable to that of the parent strain (Lizano et al., 2007).

T4 pili of an M4 non-encapsulated strain (FCT-5) promoted adherence to HaCaT cells and human nasal septum RPMI 2,650 cells, survival in human blood, and virulence in both mouse skin and peritoneal infection models (Chen et al., 2020). Other studies have showed that the major subunit of T4 pili sequesters the serum protein haptoglobin to confer M4 GAS resistance to antimicrobial peptides released by neutrophils and platelets (Köhler and Prokop, 1978; Lämmler et al., 1988; Chen et al., 2020). Binding to haptoglobin was not observed for M1 strains. Increased expression of the major subunit gene was also associated with virulence of a non-encapsulated M4 GAS strain in an intraperitoneal mouse infection model (Galloway-Pena et al., 2018).

Virulence of an M2 strain (FCT-6) was examined using a *Galleria mellonella* infection model. Survival of infected *G. mellonella* was decreased by deletion of all pilus-related genes (Tsai et al., 2017). The mutation compromised the ability to survive in both macrophage cell lines and human whole blood. The major pilin subunit bound fibrinogen, and fibrin clot formation in human plasma was partially inhibited in the presence of the recombinant major pilin.

## The Structures of Pilus Subunits and Other Proteins Encoded in FCT Region

The structures of several pilus proteins have been solved by X-ray crystallography. This has been instrumental in understanding the mechanisms of assembly and the trypsin-resistant property of pili. With pilin structures of other Gram-positive pathogens revealed, it has become evident that major and minor pilins are assembled in a modular fashion with tandem Ig-like domains of CnaA and/or CnaB domains, which are present in the *Staphylococcus aureus* adhesin Cna (Deivanayagam et al., 2002; Zong et al., 2005). Crystal structure analysis of the major subunit FctA from an M1 strain revealed that it is comprised of two immunoglobulin (Ig) folds, each of which contains a CnaB domain (Kang et al., 2007). Crystal packing of FctA showed a head-to-tail orientation, with the side chain of the lysine residue K161 adjacent to the C terminus of the next molecule (Kang et al., 2007). Mass spectrometry analysis of fragmented pili and gene mutagenesis analyses demonstrated that a covalent linkage is formed between K161 and T311 on adjacent subunits. Those residues are positioned in the omega loop of the CnaB fold and the LPXTG-like sortase recognition motif, respectively (Kang et al., 2007). Linkage between K161 and T311 allows polymerization of FctA by the class B sortase SrtC1 (Spy0129) (Barnett et al., 2004). The canonical YPKN pilin motif is not present in FctA (Ton-That and Schneewind, 2003) and the K161 position is different from that observed in major subunits of other bacterial species such as SpaA of *Corynebacterium diphtheriae* (Kang et al., 2009). The SpaA acceptor lysine in a YPKN pilin motif is located on the last β-strand of the N-terminal domain, close to the junction between domains, whereas the location of the acceptor lysine of FctA is near the top of the N-terminal domain. Thus, it is speculated that the difference in position of the acceptor lysine is correlated with type of pilus-specific sortase, i.e., class B or class C (Kang et al., 2011).

In an earlier study, Kang et al. (2007) also uncovered a striking characteristic feature of Gram-positive pilus subunits, namely formation of intramolecular isopeptide bonds between side chains of lysine and asparagine. Unlike the sortase-mediated linkage between subunits, this isopeptide bond is autocatalytically formed close to the domain boundary by an intramolecular reaction that involves a glutamic acid residue and surrounding aromatic residues. One intramolecular isopeptide bond is formed in each of the two CnaB domains (K36-N168 in the N terminal domain and K179-N303). A lysine residue in the first β-strand is linked to an asparagine residue in the last β-strand, endowing the pilin with thermal stability, resistance to proteolysis and mechanical stress (Kang and Baker, 2009; Alegre-Cebollada et al., 2010). Also, an intramolecular isopeptide bond occurs in the minor pilus subunit Cpa and in other Gram-positive bacterial Antigen I/II family of proteins (Forsgren et al., 2010; Hagan et al., 2010; Larson et al., 2011; Walden et al., 2014).

A minor subunit located at the pilus tip is considered to play a critical role in binding to host cells because of its positional advantage to reach the cell surface. The tip protein in FCT-2, FCT-3, and FCT-4 strains is Cpa (Figure 2). The C terminus (carboxyl group of C-terminal threonine residue) of Cpa (Spy0125) is linked to the above-mentioned lysine residue (K161) responsible for intermolecular linkage of FctA. Examination of the crystal structure of the Cpa C-terminal region (SPy0125, N286-T723) from an M1 strain revealed a three-domain structure, two of which contain an intramolecular isopeptide bond, K297-D595 and K610- N715 (Pointon et al., 2010). Moreover, an unusual thioester bond is internally formed between the side chains of a cysteine and a glutamine residue. This type of thioester bond has only been reported in proteins of the immune system, such as complement C3 and C4, complement-like proteins, and α2-macroglobulin (Chu and Pizzo, 1994; Law and Dodds, 1997; Cherry and Silverman, 2006), suggesting potential for covalent binding of Cpa to host factors. As in the case of FctA, intramolecular isopeptide bonds contribute to resistance to proteolysis and thermostability, whereas an alteration affecting the thioester bond had less influence on protein stability (Walden et al., 2014). Prevention of the thioester from Cpa compromised the ability of an M1 strain to bind to HaCaT cells, indicating a direct role in interaction with the host (Pointon et al., 2010). Linke-Winnebeck et al. (2014) reported that the N-terminal domain of Cpa (CpaN) from an *emm* ST6030.1 strain contains an additional thioester bond. X-ray crystallography and mass spectrometry analyses found that CpaN forms a dimer cross-linked by a polyamine spermidine, which was derived from *Escherichia coli* during recombinant protein preparation. They also reported that both thioesters contribute to binding to spermidine. This indicates that the reactive thioester has a preference for amine groups although the mechanism of covalent receptor binding has yet to elucidated.

SfbI/PrtF1 and PrtF2/FbaB are fibronectin binding proteins whose genes are located in the FCT genomic region that contain N-terminal domains homologous to the thioester-containing domain of Cpa. A homology search using the domain revealed that similar thioester domains are also present in diverse Gram-positive bacterial cell wall proteins, suggesting that the reactivity of thioester bonds is exploited by other surface proteins in other pathogens (Linke-Winnebeck et al., 2014; Walden et al., 2015). SfbI/PrtF1 also binds to the A subunit of fibrinogen in a thioester-dependent manner (Walden et al., 2015). Together, these studies provide a paradigm shift in understanding interactions between host and pathogens. It is likely that Gram-positive bacterial adhesins evolved to use covalent binding to host cells. Future studies are needed to determine the host target molecules of pilus adhesins, such as Cpa, which may provide information about GAS tissue tropism and provide the basis for effective therapeutic intervention.

The crystal structure of the minor subunit FctB from a T9 strain (Linke et al., 2010) comprises Ig-like and proline-rich tail domains and has no intramolecular isopeptide bond. The lysine residue responsible for linkage to FctA resides in the final β-strand of the Ig-like domain. The LPXTG motif of FctB in an M1 strain is LPSTG while the LPXTG-like tripartite motif (LPLAG) was found in other serotypes (Janulczyk and Rasmussen, 2001). This motif is recognized by the house-keeping sortase SrtA and promotes cross-linking to an alanine residue in cell wall peptidoglycan. The incorporation of the basal pilin into growing pili halts polymerization of FctA. Thus, all pilus components are connected by isopeptide bonds and finally become anchored to the cell wall.

Although the exact function of SipA/LepA remains unknown some predictions can be made from structural and biochemical data previously reported by Young et al. (2013; 2014b). SPase-I and SipA/LepA have a peptide-binding groove. In the crystal packing of SipA/LepA, the peptide-binding groove of one molecule is associated with the N-terminal peptide chain of the other molecule, indicating an ability of SipA/LepA to bind peptides. Young et al. also performed pull-down assays with recombinant FctA containing extracellular regions of both the signal-peptide and sortase motif, but no association of SipA/LepA and FctA was noted. Additionally, there was no interaction between SipA/LepA and the pilus-specific sortase. The same was true for synthesized peptides encompassing the extracellular region of the signal-peptides of Cpa, FctA, and FctB and the sorting motif region of FctA. The authors speculated that no detectable association was attributable to the non-physiological octameric structure of recombinant SipA and a possible requirement of the membrane-spanning region for fully functional SipA/LepA, as seen with SPase I (Carlos et al., 2000). Also, they speculated that SipA/LepA might recognize sorting signals of pilus subunits coordinately with SrtC, or provide a scaffold that modifies or deploys pilin proteins for SrtC enzymatic activity. Thus, the interactions between SipA/LepA and pilus-related factors remain unclear and further exploration is needed.

Finally, the crystal structure of SrtC1 (class B family) has also been reported (Kang et al., 2011). SrtC1 has a canonical sortase fold, in which 8-stranded β-sheets mainly in C-terminal regions generate a core β-barrel, with the surface modified with loops and helices. This β-barrel structure has a concave surface that provides an active site comprising the key catalytic residues Cys, His, and Arg. Differences occurred in conformation of β4/β5 and β7/β8 loops between two molecules in the crystal, which suggested a level of flexibility important for SrtC1 function. The catalytic residue His126 located at the start of the β4/β5 loop was positioned differently in the two molecules in the crystal, thus potentially enabling a dual acid/base role by protonating the leaving group in the cleavage reaction and deprotonating the attacking amine in the transfer reaction (Suree et al., 2009). The corresponding region of *B. anthracis* SrtA is also flexible (Weiner et al., 2010). Conformational flexibility also occurs in the β7/β8 loop of *B. anthracis* and *S. aureus* SrtA, which may be a lipid II-binding site (Suree et al., 2009; Weiner et al., 2010). The length and conformation of the β7/β8 loop is highly variable among different sortases, suggesting a role in binding the second substrate. The structure of SrtC1 is closely related to class B sortases from *S*. *aureus* and *B. anthracis*, which anchor NPQTN motif-containing surface proteins to the cell wall, and those share the same surface loops and helices (Zhang et al., 2004; Zong et al., 2004; Kang et al., 2011). On the other hand, the unique characteristics of pneumococcal pilus-specific class C sortases, including flexible lids and a C-terminal transmembrane region, were not observed in SrtC1. It was concluded that the pilus polymerizing activity is a consequence of the co-evolution of the pilin and the cognate sortase, thus enabling substrate selection (Kang et al., 2011).

It is not clear why FCT-2, FCT-3, and FCT-4 GAS use a class B sortase and SipA/LepA for pilus biogenesis. It may be associated with differences in domain structures (two domains in FctA compared to three to four domains in others) and positions of the nucleophilic lysine residues for intermolecular linkage. Additional biochemical and biophysical analysis of the interactions of pilin, SipA, and SrtC are needed to unravel the exact mechanism of assembly of these pili. Additionally, analysis of other FCT-form pili will provide insights regarding the biological consequences of GAS pilus diversity.

## Prospects for a Pilus-Based Vaccine

No vaccines are currently available for GAS. The M protein has been proposed as a primary vaccine candidate since IgG reactive to the hypervariable N-terminal region induces complement deposition and phagocytosis (Jones and Fischetti, 1988). Thus, construction of N-terminal peptide chimeras from multiple M proteins formed 26- and 30-valent M protein-based vaccines (Steer et al., 2009a; Dale et al., 2013). These were designed to provide coverage against strains circulating in developed countries. They also exhibit protection against some strains expressing M proteins that are not included among the targets of the vaccine (Dale et al., 2013). However, efficacy remains uncertain in countries where circulating strains exhibit a high level of diversity (Steer et al., 2009b).

There are 21 known T serotypes. While any protective effects of antibodies in T-type specific serum have not been reported, vaccination with recombinant pilin proteins can be effective, as noted below. T-typing serum is directed to trypsin-digested pili and it is likely that epitopes for typing are not necessarily equivalent to those exposed on the surface of native pili. One possible reason is that different epitopes exposed by trypsinization are responsible for T typing. Development of a pilus-based vaccine would be beneficial since there is less variation of T-antigenicity than observed with M-antigenicity or *emm*-typing, and fewer antigens would provide comparable coverage. Faulgi et al. sequenced *tee* genes of 39 strains representing 23 *emm* types and classified the *tee* genes into 15 clusters, with a sequence identity of greater than 90% within each cluster. The authors suggested that a vaccine containing epitopes from 12 types of T antigens would provide 90% coverage in the United States and EU (Falugi et al., 2008). Thereafter, the *tee* genotype was extended to 18 types and six subtypes, and it is expected that protective epitopes from these 18 T antigens could provide nearly full coverage for globally disseminated strains (Steemson et al., 2014). Individual *tee* alleles are highly stable over time and among geographical locations, further supporting T antigens as suitable vaccine candidates. Moreover, pili protrude from the bacterial surface by up to 2 μm (Mora et al., 2005; Kang et al., 2007), leading to unimpeded accessibility by the immune system and allowing exposure of many epitopes. Recently, whole-genome sequence analyses of 1,454 invasive GAS strains in the United States showed that 1,388 (95.5%) had one of the 21 different pilus (*tee*) types (Chochua et al., 2017).

Immunization with pilus subunit proteins confers protective immunity in mouse infection models (Mora et al., 2005; Loh et al., 2017). When serum reactivity against pilus components was tested using 100 serum samples obtained from children recovering from GAS pharyngitis using a protein array carrying four kinds of major pilin subunits and seven minor subunits, 76 of the samples reacted with at least one pilin protein (Manetti et al., 2007). Also, IgG in five of six serum samples from acute rheumatic fever patients reacted with T6 (Young et al., 2014a). Those studies indicated that pili are produced *in vivo* during infection and elicit specific antibody responses which supports their relevance as vaccine targets. However, since invasive GAS strains of FCT-3 may not produce pili *in vivo* at the inner body temperature of 37°C, a potential drawback of a pilus-based vaccine is lack of effectiveness for a subset of strains. Furthermore, it is not clear whether vaccination with pilus-based antigens generates autoantibodies, which was problematic with M protein-based vaccines.

An important concern related to development of a type-specific epitope-based vaccine is the potential of the bacterium to generate new epitopes by intragenic recombination leading to loss of opsonizing ability of antibodies directed toward this region (Jones et al., 1988). This has not been reported thus far for pilus genes. However, the FCT region is a recombination hotspot. The possibility that interaction with the immune system induces antigenic drift should be examined.

Since a major site of GAS infection is the mucosal surface of the upper respiratory tract, generation of a mucosal immune response might be important for providing protection against infection (D’Alessandri et al., 1978; Batzloff et al., 2005). The food-grade organism *Lactococcus lactis* has been used as a vehicle to deliver vaccine antigens without adjuvants to the mucosal surface and elicits immune responses in animal models (Robinson et al., 1997). *L. lactis* has also been tested for delivering mucosal vaccines against pili (Buccato et al., 2006). Immunization of rabbits with a heat-killed *L. lactis* strain expressing either FCT-3 or FCT-4 pili via the oral gavage elicited specific antibody responses (Loh et al., 2017). Anti-pilus antibodies inhibited bacterial adhesion and immune serum efficiently promoted opsonophagocytic killing of bacteria. The authors speculated that the T antigen was the most likely target for opsonophagocytic killing. Furthermore, intranasal immunization of mice with a pilus-expressing *L. lactis* strain also improved clearance rates of GAS following nasopharyngeal challenge. These results demonstrate the potential for a pilus-based vaccine to protect against GAS infection.

Exploitation of the pilus biogenesis system and *L. lactis* has been utilized to present non-pilus related antigens (Quigley et al., 2010; Chamcha et al., 2015). The *E. coli* maltose-binding protein (MBP) was fused to the C-terminal region of the pilus tip protein (Cpa) of GAS T3 pili and expressed with pilus genes in *L. lactis* allowing the MBP to be presented on the tip of pili. This strain induced both systemic and mucosal responses against the MBP. Localization of a vaccine antigen on the pilus tip and covalent fixation to the lactococcal cell wall may be an effective strategy to promote exposure of vaccine antigens, though the influence of pilus-biogenesis factors on immunization must be considered.

The group of Thomas Proft developed PilVax, another vaccine platform that uses the GAS pilus and *L*. *lactis*. Immunogenic peptides were inserted into 3 different loop regions of FctA, resulting in assembly of pili and presentation of multiple peptides on the surface of *L*. *lactis*. Mouse intranasal immunization was shown to elicit both systemic and mucosal responses (Wagachchi et al., 2018; Clow et al., 2020).

## Biotechnological Applications of FCT Proteins

Covalent linkage between proteins has been employed in therapeutics, biomaterials, diagnostics, and vaccines (Reddington and Howarth, 2015). Several methods have been used to generate stable protein complexes with diverse features related to efficiency, specificity, and stability (Rashidian et al., 2013; Antos et al., 2016; Stevens et al., 2016). For example, a cross-linking method based on an intramolecular isopeptide bond of FctA from an M1 strain has been reported (Zakeri and Howarth, 2010; Abe et al., 2013). FctA was split into two fragments at the final b-strand, with one fragment (pilin-C, residues 18–299) containing the reactive K179 and the other termed isopeptag consisting of 16 amino acids including reactive N303. These two fragments spontaneously formed an isopeptide bond *in vitro* and in *E. coli* as well as on the surface of mammalian cells. The reaction yield and rate were independent of temperature (range 4–37°C) and pH (range 6–8) in several conventional buffer systems, thus highlighting numerous advantages over other methods (Zakeri and Howarth, 2010).

Attempts of applying engineered FctA of an M1 strain as a protein shackle have also been reported (Matsunaga et al., 2013). By utilizing the intramolecular isopeptide linkage of FctA, the molecule could spontaneously polymerize into nanochains under reductive conditions. Later, the Howarth laboratory investigated the feasibility of exploiting fragments of other proteins containing isopeptide bonds. The SpyCatcher-SpyTag system is based on the isopeptide bond formed in the CnaB2 domain (K31-D117) of FbaB of FCT-3 strains (Terao et al., 2002; Hagan et al., 2010; Zakeri et al., 2012). The CnaB2 domain was split into two fragments, a 13-residue peptide from the C-terminal β-strand containing reactive D117 (SpyTag), and the rest of the 138-residue fragment termed SpyCatcher containing the reactive K31 and catalytic E77. Those fragments formed a covalent bond with high affinity under a wide range of conditions (Zakeri et al., 2012). A distinct tag-catcher system, SdyTag-SdyCatcher, has also been engineered from the CnaB domain of a fibronectin-binding protein from *S*. *dysgalactiae* (Tan et al., 2016). Subsequently, the SpyLigase-SpyTag-KTag system was developed from the SpyCatcher-SpyTag system (Fierer et al., 2014). Briefly, SpyCatcher was split into two factors, the scaffold protein SpyLigase, and the 10-residue peptide KTag containing catalytic E77 and K31, so that the active lysine, aspartic acid, and catalytic glutamic acid residues could be separately distributed into three factors. A mixture of the three factors generated a linkage between SpyTag and KTag, though the formation was dependent on strict buffer conditions and low temperature (Veggiani et al., 2016). The SpyCatcher-SpyTag system was then further modified with a phage display selection as well as more rational design to reach an improved affinity, and applied to the Spy&Go protein purification system with a non-reactive SPyCatcher mutant (SpyDock) for affinity purification of Spy-tagged proteins (Keeble et al., 2019; Khairil Anuar et al., 2019; Keeble and Howarth, 2020).

Similar systems have also been developed using the D4 domain of the pneumococcal pilin RrgA (Izoré et al., 2010; Veggiani et al., 2016). This domain contains an isopeptide bond formed between K742 and N854, which is catalyzed by the adjacent catalytic residue E803. Two fragments, SnoopCatcher and the 12-residue peptide SnoopTag, are generated, which contain N854/E803 and K742, respectively. This system can be simultaneously used with the SpyCatcher-SpyTag system (Veggiani et al., 2016). The SnoopCatcher-SnoopTag system was also developed into the SnoopLigase-SnoopTagJr-DogTag three-component system (Buldun et al., 2018), and showed a higher level of efficiency and required less strict buffer conditions as compared to the SpyLigase-SpyTag-KTag system. SpyCatcher-SpyTag and related systems have been used in a wide variety of applications, including protein labeling, stable and directional protein display on surfaces and particles, modular covalent assembly with scaffolds of multimeric structures of other proteins, and increasing enzyme resilience by cyclisation (Keeble and Howarth, 2020), as well as for the study of bacterial proteins (Hatlem et al., 2019). These systems can also be used in GAS research, such as generation of modular vaccine antigens, and promoting protein complexes for structural and functional analyses.

An attempt has also been made to introduce an isopeptide bond into a non-isopeptide-containing protein. Kwon et al. introduced lysine, glutamic acid, and asparagine residues, N13K, Q67E, and P117N, respectively, into rational positions in the non-isopeptide-containing CnaB-type fold of FctB, with one more change (V26F) that restricted movement of the engineered lysine residue to bring it closer to N117 and entrap it in a hydrophobic environment. Spontaneous formation of an isopeptide bond was observed and thermal stability was increased. This method for stabilizing IgG-like proteins could be adopted for engineering of antibodies that share similar β-clasp Ig-type domains (Kwon et al., 2017; Young and Baker, 2020).

## Discussion

Recent research with streptococcal pili has revealed the diversity of structure, function, and control of expression and revealed their potential as vaccines antigens. The revelation that the major pilin subunit is the T antigen underscored the importance of pili as an epidemiological marker. Combined with epidemiological and evolutionary studies, analyses of the diversity of the genetic organization of the FCT region indicate a relationship of its components with tissue tropism (Bessen, 2016). Furthermore, unexpected finding of an intramolecular isopeptide bond allowed development of tools with a wide range of applications (Keeble and Howarth, 2020). However, important pilus-related issues with regard to clinical and biological consequences await experimental confirmation.

Important questions have arisen related to pilus binding partners. In consideration of the positional advantage over other MSCRAMMs, primary contact with host molecules and interactions with host cells might be initiated by pili. In other words, bacteria may evolve to locate an adhesin at the tip of a long shaft, despite pilus synthesis being an energy-consuming process. This has inspired a hypothesis where host cell adherence determines both host specificity and tissue preference (Bessen, 2016). An especially intriguing question is which host molecules are targets of the thioester-containing domain (TED)-mediated linkage of the pilin adhesin Cpa. Binding partners for some pilin proteins have been reported although it is unclear if those interactions confer tissue specificity. Although TED-mediated binding of the fibronectin-binding protein PrtF1/SfbI to the human fibrinogen Aα subunit has been revealed, notable differences seen between structures of TED from various molecules hint at the presence of target specificity (Walden et al., 2015). The most prominent differences in Cpa proteins from different serotypes lie in the N-terminal region that contains a TED domain, which also raises the possibility of variations in ligands or binding affinity (Kreikemeyer et al., 2005). To clarify the relevance of TED-mediated binding for GAS pathogenesis, further research is needed for identification of binding partners of Cpa as well as other adhesive pilins whose ligands remain unknown.

Despite remarkable advances in structural analyses of pilin and related factors in several different bacterial species it is not well understood how Gram-positive pilus assembly is spatially and temporally organized on the cell wall. Detailed knowledge of the structures of protein complexes, such as the sortase/full-length pilin complex, will be required to gain insight into the functionality of sortases, including substrate specificity. Furthermore, SipA/LepA only exists in FCT-2, FCT-3, and FCT-4, with the class B sortase SrtC and requirement of SipA/LepA for pilus assembly in certain FCT forms remains to be addressed. It has been speculated that the function of SipA is recognition of pilin sorting signals in a coordinated manner with SrtC or that it constitutes a scaffold that positions pilin proteins for optimal sortase transpeptidase activity (Young et al., 2014b). Solving the structure in complex with full-length pilin and detailed mutagenesis analyses are expected to reveal the function.

The regulation of pilus biogenesis is complex and occurs at several different levels. Temperature dependent regulation is important in FCT-3 strains. This is primarily governed by post-transcriptional control of *nra* mRNA translation via a stem loop structure in the coding region (Nakata et al., 2020). The stem loop is positioned proximal to the ribosome border and is considered to be important for promoting translation, although other factors such as its distance from the translation start codon and other mRNA structures around the Shine and Dalgarno sequence may contribute. This type of translational regulation leads to the hypothesis that larger and likely specific subsets of the GAS mRNA repertoire is thermoregulated during the initial stage of infection in the throat and skin, where the temperature is lower than core body temperature. Such information may provide insight into the contribution of pili to tissue tropism. Differences in the mechanisms of controlling pilus gene expression between FCT-3 and other forms may be attributable to variations in pilus functions, such as sensitivity to the human immune system and relative contribution to virulence and fitness in the host. In other FCT forms, *nra* is replaced with *rofA* and molecular epidemiology suggests that these genes have undergone balancing selection (Bessen et al., 2005).

A long-standing goal of GAS research is development of an effective vaccine. Clinical trials of a multivalent M protein vaccine have been conducted (Kotloff et al., 2004). Along with factors extracted by use of a population-derived sequence approach (Davies et al., 2019) and antigens shown to be effective in animal studies (Azuar et al., 2019), T antigens have also been demonstrated to be viable vaccine antigen candidates. Unlike a multivalent M protein vaccine, there are fewer T type variations. However, comparative structural analyses of three two-domain T antigens (FctA), including T3, T13, and T18, revealed that the overall core structure is conserved and variations are distributed through the entire region (Young et al., 2019). Ideally, a candidate vaccine antigen would comprise a multivalent linkage of whole T antigens or domains. Further comparative crystal structure analyses and examination of pilin regions for antigenicity may lead to refinement of protective epitopes and development of a peptide-based pilin vaccine. Theoretical findings of combinations of a multivalent vaccine with other antigens, such as a family of M-related proteins (Frost et al., 2020), have demonstrated increased vaccine coverage and enhanced effectiveness (Courtney et al., 2017). Such combinations with other vaccine antigens might offer potentiating effects on prophylactic efforts for combatting GAS infections.

## Author Contributions

Both authors conceived the concept for this review article, participated in writing the manuscript, contributed to reading, editing, and reviewing the manuscript.

## Conflict of Interest

The authors declare that the research was conducted in the absence of any commercial or financial relationships that could be construed as a potential conflict of interest.
